# From Oxidative Stress to Male Infertility: Review of the Associations of Endocrine-Disrupting Chemicals (Bisphenols, Phthalates, and Parabens) with Human Semen Quality

**DOI:** 10.3390/antiox11081617

**Published:** 2022-08-20

**Authors:** Irma Virant-Klun, Senka Imamovic-Kumalic, Bojana Pinter

**Affiliations:** 1 Clinical Research Centre, University Medical Centre Ljubljana, Vrazov Trg 1, 1000 Ljubljana, Slovenia; 2 Faculty of Medicine, University of Ljubljana, Vrazov Trg 2, 1000 Ljubljana, Slovenia; 3 Division of Obstetrics and Gynecology, University Medical Centre Ljubljana, Slajmerjeva 3, 1000 Ljubljana, Slovenia

**Keywords:** subfertility, BPA, oxidative stress, DNA damage, testosterone, sperm, antioxidants, epigenetics, spermatozoa, apoptosis

## Abstract

Exposure to endocrine-disrupting chemicals (EDCs) may result in oxidative stress and endocrine system disturbance, which can have an impact on human reproduction and development. In male reproductive health, EDCs have been related to impaired reproductive function and male infertility, altered fetal development, and testicular germ-cell, prostate, and breast cancers. We conducted an electronic search using PubMed on endocrine disruptors related to oxidative stress and male infertility, and evaluated their association with endocrine-disrupting chemicals (bisphenols, phthalates, and parabens) in 25 articles. Higher levels of urinary bisphenols showed correlation with impaired semen quality and increased DNA damage. Considering phthalates and their metabolites, all studies found a positive association between urinary levels of phthalates and at least one semen parameter indicative of low semen quality; some studies also revealed sperm DNA damage. The studies on parabens less often revealed correlation of urinary parabens concentrations with a decrease in sperm count, as well as motility and DNA damage. Moreover, EDCs can elevate ROS production and lipid peroxidation, increase apoptosis, induce epigenetic modifications, and change the Y:X sperm chromosome ratio and sperm protein composition. Our review revealed detrimental effects of EDCs on semen quality and sperm DNA integrity—especially in BPA and phthalates, but also in parabens.

## 1. Introduction

The term “endocrine-disrupting chemical” (EDC) has been defined as “an exogenous substance or a mixture that alters function(s) of the endocrine system and consequently causes adverse health effects in an intact organism, or its progeny, or (sub)populations” [1]. Several studies have found that the endocrine system of exposed individuals may be disrupted by EDCs, and exposure can also have an impact on human reproduction and development [2,3,4,5]. EDCs can interfere with the normal functions of endogenous hormones by changing hormone levels, blocking or boosting hormone production, or modifying hormone transport across the body, thereby disrupting hormonal control [4]. Exposure to EDCs in humans can occur via absorption through the skin, inhalation in the air, or ingestion of contaminated food and water. However, human exposure to EDCs occurs most frequently when contaminated food is consumed. Bisphenol A (BPA), phthalates, parabens, nonylphenols, and heavy metals are the EDCs most frequently found in contaminated food. In male reproductive health, EDCs have been associated with alternations in fetal development, such as hypospadias and cryptorchidism, as well as lower semen quality, resulting in male infertility. In addition, development of testicular germ-cell, prostate, and breast cancers has been observed in relation to EDCs [4,5,6].

In almost half of infertile couples, the male factor is the sole or secondary cause of infertility [7]. Numerous endocrine disruptors (EDs) have been researched for their effects on male fertility, including phthalates, polychlorinated biphenyls, dioxins, pesticides, and parabens [8,9]. According to epidemiological data, the rise in EDCs in the environment over the past 50 years has been linked to an increase in male reproductive tissues. These chemicals have been linked to a decline in semen quality and direct effects on spermatozoa, such as changes in motility, viability, and acrosomal reaction due to the generation of oxidative stress. Additionally, EDCs have been suggested as a potential factor in testicular dysgenesis syndrome [10].

Phthalates are among the many environmental chemicals and xenobiotics that have been found to cause oxidative stress, which may affect the endocrine system and result in reproductive anomalies. Oxidative stress is related to free radicals, such as reactive oxygen species (ROS), which are produced either via endogenous processes such as normal cellular metabolism, or by external factors such as radiation, chemicals, and hyperoxia. Numerous studies show that ROS are a double-edged sword: they can play a crucial signaling role in physiological processes, while also playing a role in pathological processes. The capacity of the endogenous cellular antioxidant defense system is severely overwhelmed when ROS are present at higher levels, leading to oxidative stress [11,12].

Uncontrolled ROS generation may be damaging to cells and other biomolecules, including amino acids, proteins, carbohydrates, lipids, and deoxyribonucleic acid (DNA), which can all be destroyed. ROS can have a detrimental effect on sperm function and quality [13,14,15,16] due to the spermatozoa’s decreased motility [17], DNA damage [18,19,20], and the compromised integrity of the cellular membrane [15,21,22].

### 1.1. Bisphenol A (BPA)

Bisphenols (BPs) are a significant class of EDCs. The most well-known and possibly most extensively researched bisphenol is called bisphenol A (BPA). For more than 50 years, BPA, 4,40-isopropylidenodi-phenol, 2,2-bis(4-hydroxyphenylo)propane—a crystalline chemical compound—has been widely used as a key monomer of epoxy resins and polycarbonate (PC) plastics. BPA-based PC plastics have achieved widespread success thanks to their resilience, flexibility, and durability. As a result, they are used in a wide range of industries, from the arms industry to make safety equipment (e.g., helmets), to the production of medical devices such as dental sealants and fillers [23,24,25,26]. Years ago, it was established that even plastic baby bottles contain BPA [27]. According to the major regulatory agencies, dietary exposure to BPA is the main way that people are exposed to it. A tolerable daily intake (TDI) of 50 μg/kg body weight/day has been established based on studies using rodent models, where harmful effects were clearly seen at much higher doses [24,28,29,30,31,32,33,34].

Preclinical research findings have shown the endocrine-disrupting effects of BPA on male reproductive functions, elucidating potential pathways by which BPA can interfere with the control of spermatogenesis—primarily through the hypothalamic–pituitary–gonadal axis [35]. The three primary BPA substitutes—bisphenol F (BPF), bisphenol S (BPS), and bisphenol AF (BPAF)—share homologies with BPA in terms of chemical structure, but have received far less research than BPA. However, the widespread use of BPs has made them problematic for the environment and human health. Harmful effects of BPs on humans are receiving increasing attention, but studies on their reproductive toxicity are still limited. The study of Gao et al., revealed that BPs disturbed germ cell proliferation, induced germ cell apoptosis, and perturbed sperm functions and spermatogenesis, as a result of the disruption of testosterone biosynthesis in Leydig cells [36].

### 1.2. Phthalates

Phthalates are one of the classes of environmental EDs that are used as plasticizers for polyvinyl chloride plastics to increase the flexibility, durability, and longevity of the plastics. Phthalates include monoethyl phthalate (MEP), dimethyl phthalate (DMP), mono-2-ethylhexyl phthalate (MEHP), di-2-ethylhexyl phthalate (DEHP), diisobutyl phthalate (DIBP), monobenzyl phthalate (MBzP), mono-*n*-butyl phthalate (MnBP), mono(2-ethyl-5-hydroxyhexyl) phthalate (MEHHP), mono-*n*-octyl phthalate (MnOP), monoisononyl phthalate (MiNP) and mono-2-ethyl-5-carboxypentyl phthalate (MECPP). Numerous individuals are potentially exposed to these chemicals due to widespread use of plastic products in daily life, in addition to thousands of individuals employed in the production of plastic and plastic products, as well as the recycling of plastic [37]. Although phthalates are quickly metabolized and eliminated, their widespread use ensures ongoing exposure from conception to adulthood, as shown by numerous human biomonitoring studies conducted around the world [38]. In general, urban men were found to have much higher levels of phthalate esters in their semen than rural men. Additionally, phthalates were found in semen at statistically higher amounts in infertile men than in fertile males [39,40,41].

### 1.3. Parabens

Ethyl paraben (EP), butyl paraben (BP), methyl paraben (MP), propyl paraben (PP), and isobutyl paraben (iBuP) are examples of the class of preservatives known as parabens that are frequently employed in cosmetic and medicinal products [42]. They are a group of parahydroxybenzoates or esters of parahydroxybenzoic acid—also called 4-hydroxybenzoic acid—according to their chemical makeup. Because of their bactericidal and fungicidal properties, they are widely used. They can be found in commonly used personal care items such as toothpaste, shampoos, shower gels, moisturizers, lubricants, makeup, and topical and parenteral pharmaceuticals. Additionally, they are frequently utilized as food preservatives, making it challenging to avoid them. In several in vitro and in vivo studies, parabens have been linked to weak estrogenic and anti-androgenic actions [43]. It was found that the percentage of spermatozoa with abnormal morphology and high DNA damage was significantly increased with increasing urinary paraben concentrations, while the percentage of motile spermatozoa and male testosterone levels decreased [42]. Additionally, a positive association was discovered between urine levels of BP and sperm XY18 disomy, and between urinary PP levels and chromosome 13 disomy, in men attending infertility clinics [44].

### 1.4. Mode of Action of EDCs

It is difficult to assess how different EDCs affect semen quality, and how this affects male fertility, because they might have an impact alone, in groups, or in conjunction with other harmful environmental agents [44,45,46]. In addition, the examination of EDCs’ impact on health is further complicated by the fact that there are also numerous metabolites of EDCs. At any point in their life, men are susceptible to exposure to these hazardous environmental agents. Prenatal, in utero exposure to these substances is quite concerning because it may have detrimental effect on adult health, including decreased reproductive function.

According to Axelsson et al., males in the highest prenatal exposure tertile to a single diisononyl phthalate (DiNP) metabolite (mono-(carboxy-iso-octyl) phthalate) had 4.3 mL smaller total testicular volume, 30% higher levels of serum follicle-stimulating hormone (FSH), and 0.87 mL lower semen volumes than men in the lowest tertile [46]. Men in the highest prenatal exposure tertile to one diethylhexyl phthalate (DEHP) metabolite (mono-(2-ethyl-5-hydroxylhexyl) phthalate) had 0.70 mL lower semen volume than men in the lowest exposure tertile. Mono-(hydroxy-iso-nonyl) phthalate and mono-(oxo-iso-nonyl) phthalate—two DiNP metabolites—had a linear correlation with serum luteinizing hormone (LH) levels. All of these changes could be related to the deterioration of male reproductive function.

Moreover, prenatal exposure to all EDCs is linked to a higher risk of hypospadias and cryptorchidism in newborns, as well as a lower sperm quality and a higher risk of testicular cancer in young adult males [47,48,49]. The most significant impacts of EDCs are unquestionably those that directly influence human spermatozoa, since they have an impact on how well they can fertilize human oocytes and how the embryo develops [41]. In addition, they can also affect the offspring or even subsequent generations [46,47,48,49]. Most of these direct effects are the consequence of oxidative stress. One of the signs of oxidative stress, among others, is DNA damage.

The purpose of this article was to review the literature and summarize the knowledge about the association of EDCs with human sperm quality and male fertility.

## 2. Methods

We conducted an electronic search using PubMed for studies on EDs related to oxidative stress and male infertility. The following medical subject heading (MeSH) terms, keywords, and their combinations were used: “endocrine disruptors” AND “oxidative stress” AND “male infertility”. The search was limited to trials in humans and published in the English language in the past 20 years up to May 2022.

The inclusion criteria included cross-sectional studies, cohort studies, and case–control studies. No randomized control trials were found. We excluded review articles and case reports. Each author assessed each article independently. To determine the final eligibility, the authors separately reviewed titles, abstracts, and full-text articles. The general characteristics of the studies, including journal name, article title, the first author’s name, study design, sample size, population characteristics, endocrine disruptors’ influence on male fertility, publication year, and country of study, were extracted from the studies and evaluated. Disagreements were settled through scientific discussions. All authors performed control checks between the final data utilized in the systematic review and the source articles to prevent extraction and data input errors.

The review was performed in accordance with the PRISMA (Preferred Reporting Items for Systematic Reviews and Meta-Analyses) statement [50].

## 3. Results and Discussion

### 3.1. Literature Search

**Figure 1:**
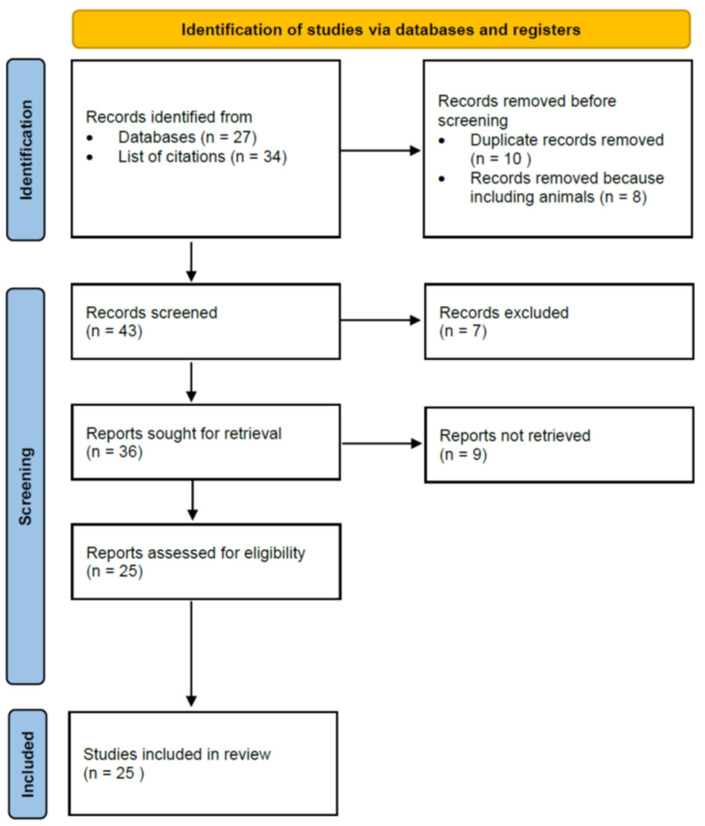
PRISMA flow diagram. Association of endocrine-disrupting chemicals—bisphenols, phthalates, and parabens—with human semen quality and oxidative stress.

The search method turned up 27 possibly pertinent articles. By reviewing the lists of citations in the articles thus obtained, we then looked for additional articles that could meet our inclusion criteria. A total of 39 publications was identified for additional full-text evaluation based on the inclusion criteria. For this review, we incorporated publications looking at correlations with bisphenols, phthalates, and parabens. Finally, we included 25 articles. Figure 1 presents a PRISMA flow diagram of the association of endocrine-disrupting chemicals—bisphenols, phthalates, and parabens—with human semen quality and oxidative stress.

### 3.2. The Association of Endocrine-Disrupting Chemicals with Human Semen Quality and Oxidative Stress

Table 1 summarizes the reported studies, with study characteristics and main findings on the effects of endocrine-disrupting chemicals—bisphenols, phthalates, and parabens—on human semen quality and oxidative stress.

#### 3.2.1. BPA, Semen Quality, and DNA Integrity

There were 10 studies identified studying the relationships between BPA, semen quality, and oxidative stress [51,52,53,54,55,56,57,58,59,60]. Some studies showed that higher levels of urinary BPA were associated with higher levels of serum male reproductive hormones (e.g., LH, testosterone) and, consequently, with impaired semen quality, e.g., sperm motility, concentration, counts, and morphology. Nevertheless, we can see that various studies are contradictory, and some of them show no a negative effects of EDCs on reproductive hormones and sperm quality. The main reason for this is probably that different groups of men were included in the studies—from young men with normal sperm quality to men with the most severe forms of infertility (for example, azoospermia)—and that the studies were cross-sectional. However, the negative effects of BPA were more evident in the groups of infertile men.

Seminal BPA, but not plasma BPA, was discovered by Vitku et al., to be inversely associated with sperm concentration, count, and morphology [53]. This finding is consistent with the recent research demonstrating that exposure to BPs is linked to a variety of adverse effects, including damage to the blood–testis barrier in the testes, a well as direct effects on spermatozoa. In addition to urine, BPs have also been found in human seminal plasma, although it is unclear how these substances might be transferred through the seminal fluid. Transport across the blood–testis barrier may be important, according to some authors.

A recent study compared the ejaculates of normozoospermic men and men post-vasectomy with interrupted vas deferens without testicular products. These men’s seminal plasma and urine were tested for BPA, BPS, and BPF concentrations. It was determined that the ratio of urinary and seminal plasma contents of BPs did not differ among men in the two groups. It was concluded that BPs are predominantly transported into seminal plasma not through testicular tissue, but through comparable ejaculate volume ratios that are applied similarly to other routes of transmission. Toxic bisphenols mostly reach the seminal plasma through the secretions of accessory glands and do so to a far greater extent than through testicular tissue [75].

Urinary BPA concentrations were discovered by Meeker et al. [51] and Omran et al. [55] to be inversely related to semen quality and positively related to DNA damage [51,55], as well as being inversely correlated with antioxidant levels [55]. As many in vitro and in vivo studies suggest that the depleted cholesterol in Leydig cells is responsible for lower testosterone production, oxidative stress may be linked to DNA damage by affecting steroid production. Additionally, the knockdown of peroxisome proliferator-activated receptor alpha (PPAR-α) remarkably ameliorated the downregulation of cholesterogenesis-related genes such as *Hmgcs1*, *Hmgcr*, and *Srebf2*, indicating that PPAR-α plays a critical role in BP-induced testicular dysfunction. Overall, these studies showed that BPS, BPF, and BPAF could have harmful effects on testicles comparable to those of BPA, and linked to the PPAR-α pathway [36].

Another crucial truth is that obesity—a common contemporary companion of the industrialized world—exacerbates the damaging effects of BPA on human spermatogenesis. Urinary BPA concentrations were shown to be inversely correlated with sperm count per ejaculate in obese males according to Hu et al., who assessed the association with semen volume, sperm count per ejaculate, sperm concentration, and sperm motility in 357 men. Furthermore, metabolomics studies were carried out to find metabolites connected to this interaction. Capric acid, dodecanoic acid, L-palmitoylcarnitine, and niacinamide were among the detected metabolites; these are known to be important in fatty acid oxidation and the tricarboxylic acid cycle, indicating higher oxidative stress linked to male reproductive dysfunction [54]. The significance of dietary exposure to BPA was supported by Caporossi et al., who found a strong positive correlation between the consumption of canned food and the levels of BPA, as well as negative correlation between the usage of plastic food storage containers and semen volume [57].

#### 3.2.2. Phthalates, Semen Quality, and DNA Integrity

We found 10 studies studying the association of phthalates and their metabolites with semen quality and oxidative stress [61,62,63,64,65,66,67,68,69,70]. All of them showed a positive correlation between levels of phthalates in urine and at least one semen parameter indicative of low semen quality. The associations were more common in groups of men with infertility than groups of fertile men. Hauser et al., revealed that low sperm concentration and motility were related to higher MBP levels [62]. Jurewitz et al., confirmed association of higher levels of urinary phthalate metabolites and lower sperm motility and testosterone levels, as well as increased sperm DNA damage and sperm aneuploidy [64]. MEHP concentrations were inversely associated with Y:X sperm chromosome ratios [68]. Bloom et al., also revealed a positive association between phthalate levels and percentages of abnormal sperm morphology—especially with regard to sperm heads [66]. Another three studies demonstrated a positive relationship of levels of urinary phthalates with sperm DNA damage [61,64,65].

According to earlier research, exposure to phthalates can impair normal spermatogenesis and steroidogenesis due to oxidative stress in the male reproductive organs—particularly in the testes and epididymis. By causing oxidative stress in germ cells or apoptosis in target Sertoli cells, they impede the spermatogenic process. Phthalates also reduce the Leydig cells’ ability to produce steroidogenic enzymes by increasing ROS. As expected, studies show that exposure to phthalate chemicals during pregnancy and postnatally may result in decreased sperm counts and other reproductive defects in the young [40]. Paternal urinary concentrations of DEHP metabolites were also correlated with higher failure rates of in vitro fertilization (IVF) treatment [41].

#### 3.2.3. Parabens, Semen Quality, and DNA Integrity

There were five studies identified studying the association of parabens with semen quality and oxidative stress [42,71,72,73,74]. Only two studies revealed the correlation of urinary paraben concentrations with a low sperm count and decreased motility [42,74]. In addition, Jurewicz et al., found a strong correlation between urinary paraben concentrations and an increase in the abnormal morphology percentage and DNA fragmentation of spermatozoa, as well as a decrease in serum testosterone levels [42]. In studies where no correlation was revealed, there was temporal intraindividual variability [71], or the sample size was small [72], or the average age of men was low, suggesting that in young men, urinary parabens may not negatively affect their reproductive function [73].

### 3.3. EDCs, Oxidative Stress, and Human Spermatozoa

**Figure 2:**
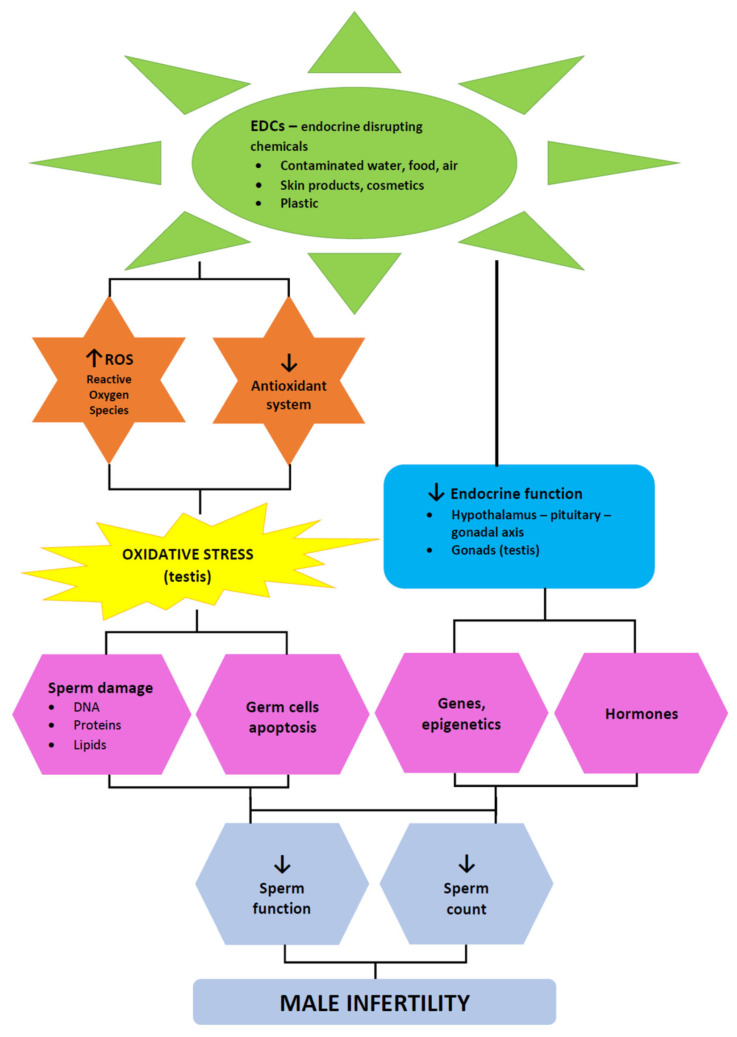
Mechanisms of action of endocrine-disrupting chemicals (EDCs) on male fertility.

Our review of the correlation of EDCs wih semen quality and oxidative stress suggests that negative associations were more frequently related to BPA and phthalates, and less frequently to parabens. The question remains—what are the possible mechanisms of action of EDCs on human spermatozoa? Figure 2 presents the mechanisms of action of EDCs on male fertility.

EDCs may be able to bind to receptors on human spermatozoa and change sperm functions, according to mounting evidence [76]. The interaction between estrogenic compounds and nongenomic receptors for estrogen on human sperm membranes has been demonstrated. The nongenomic receptor for estradiol (E2), which is located on the sperm plasma membrane, mediates the effects of this hormone on sperm’s intracellular calcium concentrations and biological response to progesterone (P). It was shown that 17βE2 expresses an inhibitory effect on P-mediated calcium influx and acrosome reaction (AR) in human spermatozoa. The anti-estrogens tamoxifen (Tx) and ICI 164,384 (ICI) induced only a slight increase in calcium influx; however, as in the case of 17βE2, this resulted in a reduction in P-stimulated calcium influx. Both agents reduced the calcium response to 17βE2 without affecting 17βE2-induced inhibition of the calcium response to P. Tx alone did not affect spontaneous or P-stimulated AR, but partially reverted the inhibitory effect of 17βE2. These results indicate that both estrogens act as agonists of the membrane estrogen receptors of human spermatozoa. On the other hand, the xenoestrogen BPA and octylphenol polyethoxylate (OP) did not express any direct effect on calcium fluxes or AR in human spermatozoa, whether in basal conditions or in response to P challenge. Although it has been suggested that environmental estrogens can imitate the actions of estrogen in other cells—perhaps by acting through genomic receptors—in human spermatozoa they have not been shown to tamper with 17βE2′s binding to its membrane receptor or the short-term effects of this hormone. These data indicate that in human spermatozoa the membrane receptor for E2 differs from the genomic receptor in terms of both biochemistry and pharmacology [77].

EDCs affect human spermatozoa in different ways:

(a) Sperm mitochondria: The study of Skibińska et al. showed that human sperm mitochondria are targets for both 17βE2 and EDCs. Two-hour incubation of spermatozoa with E2, genistein, and BPA did not alter the cell vitality or stimulate phosphatidylserine membrane translocation in spermatozoa. However, incubation of spermatozoa with E2 or BPA separately, as well as incubation with the three ligands together, altered the mitochondrial membrane potential. Incubation of spermatozoa with all three ligands significantly increased the mitochondrial superoxide anion levels in the spermatozoa. The reaction to the mixture of 17βE2 and xenoestrogens including BPA suggests a synergistic mechanism of action. Moreover, xenoestrogens may increase the sensitivity of spermatozoa to 17βE2 [78]. In another study, exposure of suspensions of motile human spermatozoa to different BPA concentrations for 4 h led to a reduction in mitochondrial membrane potential. It was also associated with an increased mitochondrial generation of superoxide anions, caspase-9 and caspase-3 activity, and decreased motility. Decreased sperm motility was observed at BPA ≥ 400 μM. Twenty hours of exposure of spermatozoa to 300 μM BPA resulted in a significant decrease in sperm vitality associated with complete sperm immobilization. Finally, 300 μM BPA also produced significant oxidative DNA damage, as revealed by the formation of the oxidized base adduct 8-hydroxy-2′-deoxyguanosine. It was concluded that BPA has an impact on the integrity of human sperm by causing pro-oxidative/apoptotic mitochondrial malfunction [79]. Interestingly, it was found that human spermatozoa are shielded against mitochondrial failure caused by exposure to BPA by an aqueous extract of the Eruca sativa plant [80]. Due to their lack of impact on the viability, motility, and mitochondrial activities of human sperm, BPS and BPF appear to be safer substitutes for BPA [81]. These findings might aid regulatory agencies in identifying safer compounds to replace BPA in the manufacture of plastics on a large scale.

Human spermatozoa exposed to the phthalates DEHP and MEHP did not exhibit any changes in their motility, viability, membrane integrity, mitochondrial activity, or homeostasis of reactive oxygen species [82]. On the other hand, a negative correlation was established between semen phthalate (DEHP) levels and sperm quality, along with positive associations with depolarized mitochondria, elevated ROS production, and lipid peroxidation [39]. Moreover, mitochondrial DNA copy numbers were positively associated with urinary concentrations of the phthalate metabolite monocarboxyisononyl phthalate (MCNP) after adjusting for age, body mass index, current smoking, race, and measurement batch in a cohort of men included in an IVF program [83]. It was proposed that the key to determining how parabens contribute to a decline in male reproductive capacity is through their interaction with mitochondrial function in the testes [84].

Furthermore, it was found that parabens also generated ROS in human spermatozoa. It was shown that combinations of parabens may induce the production of cytosolic and mitochondrial ROS in vitro, reducing sperm motility and viability in a dose-dependent manner [43].

(b) Sperm DNA fragmentation: Various studies have shown that estrogenic compounds cause oxidative stress and damage to DNA integrity—such as DNA fragmentation—in human spermatozoa. Different estrogens and estrogen analogues, including 17βE2 and BPA, increased sperm redox activity, which was linked to a severe reduction in spermatozoa motility and an increase in DNA fragmentation [85]. According to Meeker et al., there were decreases in sperm concentration, motility, and morphology, as well as an increase in sperm DNA damage, related to higher BPA urinary levels [51]. In another study, total BPA levels in the urine of 50 infertile men were found to be inversely correlated with semen quality and antioxidant levels, and positively correlated with sperm DNA damage and seminal plasma lipid peroxidation [55].

The study of Pant et al., revealed a negative correlation between semen phthalate levels (DEHP) and sperm quality, and a positive association with sperm DNA fragmentation was also established [39]. The concentration of DEHP in the seminal plasma was correlated positively with the ROS level and sperm DNA fragmentation index (DFI), but negatively with the percentage of progressively motile sperm [86]. Exposure of human spermatozoa to the phthalate DEHP in vitro significantly increased the sperm DNA fragmentation [87].

Moreover, a relationship between parabens’ potential to activate ROS production and cause oxidative DNA damage in spermatozoa and the length of their alkyl chains was discovered. At concentrations used clinically, methylparaben reduced the motility and cell viability of human sperm while increasing the generation of ROS and oxidative DNA damage [43].

(c) Apoptosis or programmed cell death: Regulating apoptosis in germ cells is essential for preserving regular sperm production. Numerous studies have demonstrated that EDCs—especially BPA—cause sperm production to decline and induce germ cell apoptosis. Activation of ADAM17 and p38 MAPK is involved in the BPA-induced apoptosis of male germ cells [88]. The exposure of human motile sperm suspensions to scalar BPA concentrations for four hours produced an increase in caspase-9 and caspase-3 activation, along with impairment of motility [89].

Some studies have suggested that, similarly to BPs, exposure to phthalates may induce sperm apoptosis. The evidence was provided that phthalate exposure in humans was linked to higher protamine levels in the spermatozoa, which may play a role in sperm apoptosis [90]. The study of Marchiani et al., showed that after in vitro incubation of human spermatozoa with phthalate, sperm’s progressive motility was dramatically reduced. Moreover, exposure to this toxic agent also induced spontaneous sperm acrosome reaction, reduced the physiological response to progesterone, and increased caspase activity, all of which suggest triggering of an apoptotic pathway [91]. It was also found that apoptosis-related gene polymorphisms might contribute to the effects of phthalate exposure on male reproductive health [92].

Furthermore, parabens can generate mitochondrial ROS in human spermatozoa, related to increased apoptosis and reduced vitality of the spermatozoa, along with decreased sperm motility [43,93].

(d) Sperm epigenetics: Methylation of long interspersed nucleotide elements (LINE-1) is a marker of genome-wide methylation status in cells. It was discovered that BPA exposure is linked to LINE-1 hypomethylation in human spermatozoa. Sperm LINE-1 methylation levels were considerably lower in BPA-exposed workers compared to unexposed individuals, according to a study by Miao et al. Additionally, log-transformed urine BPA levels were found to be negatively correlated with sperm LINE-1 methylation by linear regression analysis. Interestingly, this was not found for peripheral blood cells. Due to exposure to BPA, it was determined that these findings might have an impact on male reproductive health [94]. Similarly, Zheng et al., found genome-wide changes in DNA hydroxymethylation in the sperm of Chinese males who had been exposed to BPA. Some of the BPA-affected genes may be involved in the response to DNA damage in spermatozoa brought on by BPA. These findings also indicate that exposure to BPA affects DNA hydroxymethylation in a manner that is dependent on trimethylation of H3 in human spermatogenesis which, in turn, interferes with gene expression in human spermatozoa [95]. BPA exposure also induced hydroxymethylation of the acetylcholinesterase (*ACHE*) gene in exposed men [96]. This finding is quite important, because the *ACHE* gene is a sperm-expressed gene encoding the acetylcholine-hydrolyzing enzyme acetylcholinesterase, and participates in the apoptosis of different cells, including spermatozoa. In another study, men from infertile couples who underwent IVF had 131 differentially methylated regions (DMRs) in their spermatozoa, all of which were related to at least one urinary phthalate metabolite. These findings imply that paternal adult environmental factors may influence epigenetic reprogramming during spermatogenesis and influence the early-life development [97].

The results of another study conducted by the same group clearly demonstrated an unfavorable relationship between male preconception urinary phthalate metabolite concentrations and blastocyst quality during IVF, which most likely developed after genomic activation [98]. Similar negative correlations were observed between male urine phthalate metabolite concentrations and the chance of high-quality blastocysts. Such findings may have clinical and public health significance both for the couples undergoing IVF and for the general population if they are confirmed by additional research. Additionally, it has also been shown that DNA methylation plays a role in mediating the positive correlation between low-level environmental phthalate exposure and sperm motility. For instance, epigenetic modification via LINE-1 DNA methylation has been shown to have mediating effects in the relationship between DEHP exposure and sperm motility in men [99].

To the best of our knowledge, there are no such data for parabens to date.

The observations about the epigenetic effects of EDCs are very worrying, as epigenetic abnormalities—especially of the sperm—may be linked to individual development, and may be transmitted to subsequent generations.

(e) Ratio of Y- and X-chromosome-bearing live spermatozoa: In certain developed nations, but not all, a trend toward a lower ratio of male to female births has been observed recently [68]. Interestingly, it was proven that some EDCs may affect the Y:X sperm chromosome ratio [79]. The viability, motility, and capacitation of sperm were affected by all EDCs examined, including genistein (Gen), BPA, 2,3,7,8-tetrachlorodibenzo-p-dioxin (TCDD), dibromochloropropane (DBCP), and diazinon (Diaz). When sperm were treated with TCDD, DBCP, and Diaz, the Y:X ratio of live spermatozoa was significantly lower than it was in control spermatozoa. These findings revealed that some EDCs had a greater impact on the viability of Y spermatozoa than X spermatozoa, suggesting that a decrease in Y-carrying sperm viability may lead to offspring with a sex ratio that is disproportionately female at birth [89]. Similarly, the Y:X sperm chromosomal ratio in human spermatozoa was found to be negatively associated with EDCs such as 5OH MEHP, CDCCA to TDCCA, and 1-OHP for BPA. This suggests a relationship between the concentrations of metabolites from common environmental endocrine disruptors (e.g., phthalates, synthetic pyrethroids, polycyclic aromatic hydrocarbons) and the ratio of the sex chromosomes in human spermatozoa [68].

(f) Sperm protein composition: Using a contemporary approach of proteomics, it was discovered that BPA exposure is associated with the deregulation of proteins such as actin (ACTB), caspase-3 (CASP3), glyceraldehyde-3-phosphate dehydrogenase (GAPDHS), glutathione peroxidase 4 (GPX4), outer dense fiber protein 2 (ODF2), Ras-related protein Rab-2A (RAB2A), sperm surface protein Sp17 (SPA17), and triosephosphate isomerase (TPI1) in spermatozoa. All of these proteins play significant roles in processes related to male fertility, including sperm motility (ACTB, ODF2, GADPHS), acrosome reaction (ACTB), apoptosis (CASP3), spermatogenesis and sperm maturation (GPX4), acrosome biogenesis (RAB2A), and sperm binding to oocytes’ zona pellucida (SPA17, TPI1) [100].

### 3.4. Antioxidant Therapy for Subfertile Men

There is more and more research proving the negative impact of EDCs on semen quality and spermatozoa in humans, and these findings cannot be ignored. They require the attention and action of the entire society at all levels. There is a pressing question of how to avoid all of these negative influences. Prevention is certainly one of the options, both at the level of society and at the individual level. Experts in reproductive medicine also face this challenge—especially in targeting oxidative stress with antioxidant supplementation. Numerous studies on the effects of antioxidant supplements on semen quality have been conducted, some revealing positive effects of different antioxidants on different semen parameters [101], while others determining no effects [101,102].

The recent Cochrane review “Antioxidants for male subfertility” tried to answer the question of whether supplementary oral antioxidants improve fertility results in subfertile males as compared to men receiving a placebo, no treatment, or another antioxidant therapy. In a review by Cochrane authors, 18 different antioxidants were compared in 90 randomized controlled trials with a placebo, no treatment, or another antioxidant in a cohort of 10,303 subfertile men. The results of 12 small-to-medium-sized randomized controlled trials suggested that antioxidant supplements taken by subfertile males from infertile couples visiting an infertility clinic may increase the likelihood of a live birth, but the overall certainty of the evidence was very low. Evidence with a low degree of certainty indicates that clinical pregnancy rates could rise following antioxidant therapy. Overall, there is no evidence that antioxidant therapy increases the chance of miscarriage. Therefore, subfertile couples should be advised that the existing research on the efficacy of antioxidant therapy in improving male fertility is inconclusive [103]. The question of antioxidant therapy that could mitigate the toxicity of EDCs in men facing fertility problems is therefore still open, and needs to be further researched.

## 4. Conclusions

Our review revealed that some studies link increased urine BPA concentrations to lower semen quality and DNA damage; these detrimental effects of BPA were more evident in infertile men. Considering phthalates, all studies found positive associations between urinary levels of phthalates and at least one semen parameter indicative of low semen quality. In addition, increased sperm DNA damage and sperm aneuploidy were linked to higher urinary levels of phthalate metabolites. Some studies have shown a correlation between urinary paraben concentrations and a decline in sperm count and motility, as well as an increase in sperm DNA damage. Our findings show that EDCs can elevate ROS production and lipid peroxidation, increase apoptosis, induce epigenetic modifications, and change Y:X sperm chromosome ratios and sperm protein composition.

## Figures and Tables

**Table 1:** Association of endocrine-disrupting chemicals—bisphenols, phthalates, and parabens—with human semen quality and oxidative stress: review of the studies.

Study/Author	Year	Number of Patients	EDC	Outcome
BPA				
Meeker et al. [51]	2010	190 men at an infertility clinic	- Urine and semen samples - Urinary BPA concentrations - Evaluation of semen quality - Evaluation of sperm DNA damage (comet tail)	- Increased urinary BPA concentrations were associated with declines in sperm concentration, motility, and morphology, as well as increased sperm DNA damage
Lassen et al. [52]	2014	308 young Danish men from the general population	- Urine and semen samples - Urinary BPA concentrations measured using isotope dilution TurboFlow liquid chromatography with tandem mass spectrometry - Evaluation of sperm quality	- BPA concentration above the lowest quartile was associated with higher levels of serum T, LH, E2, and free T compared to the lowest quartile - Men in the highest quartile of BPA excretion had on average 18% higher total T and 13% higher E2 levels compared with the lowest quartile. - Men in the highest quartile of urinary BPA concentrations had a significantly lower percentage of progressive motile spermatozoa compared with men in the lowest quartile
Vitku et al. [53]	2016	191 men with different degrees of (in)fertility from an infertility clinic	- Blood plasma and seminal plasma - Determination of BPA in blood and seminal plasma	- Seminal BPA, but not plasma BPA, was negatively associated with sperm concentration, count, and morphology
Hu et al. [54]	2017	357 obese men	- Urine and semen samples - Determination of urinary concentrations of BPA - Evaluation of semen quality	- Urinary BPA concentrations were significantly correlated with sperm counts per ejaculate
Omran et al. [55]	2018	100 men: 50 infertile men and 50 control men with normal semen quality	- Urine and semen samples - Determination of urinary concentrations of BPA - Evaluation of semen quality - Evaluation of antioxidant levels and sperm DNA damage	- Urinary BPA concentrations were negatively associated with semen quality and antioxidant levels, and positively correlated with DNA damage
Adoamnei et al. [56]	2018	215 healthy men, aged 18–23 years, recruited in southern Spain	- Urine, semen, and blood samples - Determination of urinary BPA concentrations using dispersive liquid–liquid microextraction and ultrahigh-performance liquid chromatography with tandem mass spectrometry detection - Evaluation of sperm quality and reproductive hormone levels	- There was a significant positive association between urinary BPA concentrations and serum LH levels - Urinary BPA concentrations were significantly and inversely associated with sperm concentrations and total sperm counts
Caporossi et al. [57]	2020	105 men at an infertility clinic	- Urine and semen samples - Urinary levels of BPA and six phthalate metabolites (MEP, MBzP, MnBP, MEHP, MnOP, and MiNP) were analyzed by high-performance liquid chromatography with tandem mass spectrometry - Evaluation of associations between BPA and phthalates and semen quality using regression analysis	- Semen volume was positively associated with BPA, MnBP, and MnOP levels, while it was negatively associated with MiNP levels - Sperm concentration had a significant inverse relationship with MEP levels. - Negative association was found between the use of plastic containers for food storage and semen volume - Significant positive correlation between the consumption of canned food and the levels of BPA, and between the use of perfumes and levels of MEP
Palak et al. [58]	2021	116 men: 20 infertile men with non-obstructive azoospermia, 46 infertile men with oligoasthenoteratozoospermia, and 50 control normospermic men	- Semen (seminal plasma) and blood samples - Analysis of BPA in seminal plasma - Analysis of sperm quality	- Levels of BPA in the seminal plasma of azoospermic men were significantly higher compared to the healthy controls - Concentrations of E2 and A were significantly decreased in the seminal plasma of azoospermic men compared to the normospermic men - Levels of BPA were negatively correlated with sperm concentration and normal semen morphology - BPA was correlated with the miR-let-7a, miR-let-7c, and miR-518f levels in seminal plasma, suggesting that BPA may act directly in seminal plasma, affecting the testicular environment
Benson et al. [59]	2021	556 young adult Danish men, aged 18–20 years	- Urine and semen samples - Determination of urinary BPA, BPF, and BPS concentrations - Evaluation of semen quality: - volume, sperm concentration, total sperm count, sperm motility, and sperm morphology - Associations between urinary bisphenol levels and semen characteristics were estimated using an adjusted negative binomial regression model	- No associations between urinary bisphenol concentrations and semen quality were found.
Chen et al. [60]	2022	984 Chinese men from an infertility clinic	- Urine and semen samples - Analyses of urinary concentrations of BPA, BPS, and BPF - Urinary measurements were associated with semen quality	- Higher BPA exposure was associated with increased odds ratios (ORs) of having below-reference sperm concentration, total sperm count, progressive motility, and total motility - Higher BPS exposure was associated with increased ORs of having below-reference progressive motility and total motility - Higher exposure to individual BPA, BPS, and bisphenol mixtures was associated with impaired semen quality. This was not observed for BPF
Phthalates				
Duty et al. [61]	2003	168 men	- Urine and semen samples - Determination of urinary concentrations of eight phthalate metabolites using high-performance liquid chromatography and tandem mass spectrometry - Evaluation of sperm DNA integrity using a neutral single-cell microgel electrophoresis assay (comet assay)	- Statistically significant positive association between urinary MEP and mean comet extent as a measure of DNA fragmentation, e.g., damage - No significant associations were found between comet assay parameters and other urinary phthalate metabolites, including MBP, MBzP, MEHP, and MMP
Hauser et al. [62]	2006	463 male partners from subfertile couples	- Urine and semen samples - Determination of urine phthalate metabolites using solid-phase extraction coupled with high-performance liquid chromatography isotope dilution tandem mass spectrometry - Evaluation of semen quality	- Dose–response relationships of MBP with low sperm concentration (odds ratio per quartile adjusted for age, abstinence time, and smoking status) and motility - There was suggestive evidence of an association between the highest MBzP quartile and low sperm concentration
Liu et al. [63]	2012	150 Chinese men of reproductive age	- Urine and semen samples - Determination of urinary MBP phthalate concentrations - Evaluation of semen quality	- Increased urinary concentrations of MBP phthalate were associated with decreased sperm concentrations.
Jurewitz et al. [64]	2013	269 men with normal semen concentration or slight oligozoospermia	- Urine, semen and blood samples - Determination of urinary phthalate metabolites - Evaluation of semen quality (sperm concentration, motility, morphology, CASA parameters) - Evaluation of sperm chromatin structure and sperm aneuploidy - Determination of reproductive hormones	- Higher levels of urinary phthalate metabolites were significantly associated with: ◦ Decreases in sperm motility (5OH MEHP, MEHP, MINP) and CASA parameters (MBP) ◦ Decreases in testosterone levels (MEHP) ◦ Increases in sperm DNA damage (MBP) ◦ Increases in sperm aneuploidy (MBzP, MBP, MEHP, MEP)
Axelsson et al. [65]	2015	314 young Swedish men from the general population	- Urine, semen, and blood samples - Determination of urinary metabolites of phthalates - Determination of reproductive hormones - Evaluation of semen and sperm high DNA stainability (HDS)—a marker of sperm immaturity	- Levels of DEHP metabolites—particularly urinary MECPP—were negatively associated with progressive sperm motility - Men in the highest quartile of MECPP concentration had 27% higher HDS than men in the lowest quartile
Bloom et al. [66]	2015	501 men from the United States—male partners in couples discontinuing contraception to become pregnant—general population	- Urine and semen samples - Determination of 14 monoester metabolites of phthalate diesters using high-performance liquid chromatography coupled with tandem mass spectrometry - Evaluation of semen quality	- Increased levels of urinary MCMHP, MEHHP, MBzP, and MNP were significantly associated with: ◦ Lower total sperm counts and concentrations ◦ Larger sperm heads ◦ Higher proportions of megalohead sperm morphology ◦ Other morphological changes of the spermatozoa - Urinary MMP and MCPP were significantly associated with lower sperm motility. - Urinary MEHP was significantly associated with higher sperm motility
Wang et al. [67]	2015	1040 Chinese infertile men from an infertility clinic	- Urine and semen samples - Determination of urinary concentrations of eight phthalate metabolites - Evaluation of semen quality	- Urinary concentrations of MBP were found to be positively associated with below-reference sperm concentrations and total sperm counts - Significant dose-dependent relationships of the urinary level of MEHP and the percentage of DEHP excreted as MEHP (%MEHP) with an increased percentage of abnormal sperm heads.
Jurewicz et al. [68]	2016	194 men aged less than 45 years, with normal sperm concentration or with slight oligozoospermia	- Urine and semen samples - Urinary phthalate metabolites were analyzed - Semen quality was evaluated - Sperm chromosome Y:X ratio was assessed by FISH	- MEHP concentrations were negatively related to Y:X sperm chromosome ratios
Thurston et al. [69]	2016	420 men from the US—fertile partners of pregnant women	- Urine and semen samples - Determination of urinary concentrations of nine phthalate metabolites in urine: MEHP, MEHHP, MEOHP, MECPP, MBP, MiBP, MCPP, MBzP, and MEP - Evaluation of semen quality	- In adjusted linear models, urinary metabolite concentrations were not associated with any semen parameters - An inverse association between MBzP concentrations and sperm motility was found
Chen et al. [70]	2017	796 male students who experienced a relocation of campuses and shifting environmental exposure	- Urine, semen, and blood samples - Determination of 13 urinary phthalate metabolites - Evaluation of semen quality and reproductive hormones	- All but two semen/hormone outcomes were associated with at least one phthalate metabolite: Decrease in sperm concentration, total sperm number, and progressive motility
Parabens				
Meeker et al. [71]	2011	190 male partners attending an infertility clinic, aged between 18 and 55 years, without post-vasectomy status	- Urine, semen, and blood samples were collected - Urine samples were analyzed for MP, PP, BP, and BPA - Associations with serum hormone levels, semen quality parameters, and sperm DNA damage measures were assessed using multivariable linear regression	- Detection rates in urine were 100% for MP, 92% for PP, and 32% for BP - No statistically significant associations were observed between MP or PP and the outcome measures - Urinary BP concentrations were not associated with hormone levels or conventional semen quality parameters, but they were positively associated with sperm DNA damage - When urinary BPA quartiles were added to the model, BP and BPA were both positively associated with sperm DNA damage - Assessment of paraben concentrations in repeated urine samples from a subset of the men (*n* = 78) revealed substantial temporal variability
Jurewiczet al. [42]	2017	315 men who attended an infertility clinic for diagnostic purposes, with normal semen concentrations	- Urine, semen, and saliva samples - Analysis of five parabens’ concentrations using a validated gas chromatography ion-tap mass spectrometry method	- Urinary parabens’ concentrations were significantly associated with an increase in the percentage of spermatozoa with abnormal morphology, increased DNA fragmentation, and a decrease in the percentage of motility and serum T levels
Nishihama et al. [72]	2017	42 male partners of couples who visited a gynecology clinic for infertility consultation	- Urine and semen samples - Analyses of urinary parabens: MP, EP, PP, and BP - Multiple regression and logistic regression analyses of associations between concentrations of urinary parabens and sperm parameters	- No significant association was found between semen parameters and urinary paraben concentrations in multiple regression analyses and logistic regression analyses
Adoamnei et al. [73]	2018	Cross-sectional study with 215 young university students (18–23 years old) recruited in southern Spain	- Urine, blood, and semen samples retrieved on a single day - Urinary paraben concentrations were measured - Reproductive hormones (FSH, T, E2, inhibin B) were measured in serum samples - Semen quality was evaluated	- Ninety-four percent of men had detectable urinary concentrations of parabens - Urinary concentrations of parabens or their molar sum were not significantly associated with any semen parameters or reproductive hormone levels
Smarr et al. [74]	2018	501 male partners of couples planning to become pregnant	- Urine and semen samples - Urinary paraben concentrations were measured - Linear adjusted mixed-effects models were used for analysis of semen parameters	- Parabens were associated with diminished sperm count and several sperm motility parameters - Hydroxylated paraben metabolites were significantly positively associated with selected semen quality parameters

Abbreviations: A, androstenedione; BP, butyl paraben; BPA, bisphenol A; BPF, bisphenol F; BPS, bisphenol S; DEHP, di-2-ethylhexyl-phthalate metabolites; DNA, deoxyribonucleic acid; EDC, endocrine-disrupting chemical; E2, estradiol; T, testosterone; LH, luteinizing hormone; EP, ethyl paraben; E2, estradiol; MBP, mono-*n*-butyl phthalate; MBzP, monobenzyl phthalate; MCMHP, mono-[2-(carboxymethyl) hexyl] phthalate; MCPP, mono-3-carboxypropyl phthalate; MECPP, mono-2-ethyl-5-carboxypentyl phthalate; MEOHP, mono-2-ethyl-5-oxohexyl phthalate; MEHP, mono-2-ethylhexyl phthalate; MEHHP, mono-2-ethyl-5-hydroxyhexyl phthalate; MEP, monoethyl phthalate; MiNP, monoisononyl phthalate; MMP, monomethyl phthalate; MnBP, mono-*n*-butyl phthalate; MnOP, mono-*n*-octyl phthalate; MNP, monoisononyl phthalate; MiBP, monoisobutyl phthalate; MP, methyl paraben; PP, propyl paraben.

## Data Availability

The data are contained within the article.
